# Knowledge and Attitude towards Probiotics among Dental Students and Teachers: A Cross-Sectional Survey

**DOI:** 10.3390/dj11050119

**Published:** 2023-05-02

**Authors:** Ksenia Babina, Dilara Salikhova, Maria Polyakova, Alexandr Zaytsev, Anna Egiazaryan, Nina Novozhilova

**Affiliations:** 1 Department of Therapeutic Dentistry, I.M. Sechenov First Moscow State Medical University (Sechenov University), 119991 Moscow, Russia; 2 Institute of Linguistics and Intercultural Communication, I.M. Sechenov First Moscow State Medical University (Sechenov University), 119991 Moscow, Russia

**Keywords:** probiotics, knowledge, attitude, dental students, academics, survey

## Abstract

This cross-sectional survey assessed the knowledge of and the attitude towards probiotics of dental students and academics at Sechenov University, Moscow, Russia. Our questionnaire consisted of 15 questions divided into 3 sections: respondents’ sociodemographic data, knowledge on probiotics, and attitude towards probiotics. The data were analyzed using the Mann–Whitney U test, Fisher’s exact test, and Spearman’s rank correlation coefficient. Out of the 658 questionnaires distributed, a total of 239 questionnaires were completed by the undergraduates, yielding a response rate of 39.6%, and 54 by the teaching staff (response rate = 100%). Most students (53.6%) and teachers (55.5%) had a fair knowledge of probiotics (*p* = 0.3135). A vast majority of dental students (97.9%) and all teachers had a positive attitude towards probiotics, with higher mean scores among academics (*p* < 0.001). A positive weak correlation was found between knowledge and attitude (Spearman r = 0.17, *p* = 0.0027). The results obtained reveal the need for more evidence-based educational trainings for university teachers and a course on probiotics to be included in the curriculum for dental students.

## 1. Introduction

The human body is a habitat for a wide range of microorganisms, with an average of 1.3 bacterial cells per human body cell [1]. The normal microbiota provides many benefits to the human body, such as regulation of the immune system, energy supply, protection against pathogens, and lipid metabolism [2]. The composition of the microflora is host-specific and changes throughout an individual’s lifetime [3]. Factors affecting the balance of the microflora include age, origin, dietary habits, environment, application of antibiotics, and certain diseases [4].

Most dental diseases, in particular dental caries [5,6,7,8], inflammatory periodontal disease [9,10], and halitosis [11,12,13], are caused by an imbalance in the oral microflora. Therefore, the replacement of pathogenic microorganisms with commensals is one of the strategies to prevent these diseases [14,15,16]. This can be achieved by using dietary supplements, i.e., oral biotics and probiotics [17].

According to the WHO, probiotics are “live microorganisms that, when administered in adequate amounts, confer a health benefit on the host” [18].

A study by Miller et al. was one of the first to investigate the ways in which various microorganisms could be used in dentistry [19]. Since then, a number of studies examining the effectiveness of oral probiotics for the prevention and treatment of dental caries [20,21,22], periodontal diseases [23,24,25], oral candidiasis [26,27], and halitosis [28,29] have been conducted. To date, numerous studies have evaluated different ways in which probiotics affect oral health: competition for food substrates and adhesion sites [30,31], changes in secretory immunoglobulin A (sIgA) levels in saliva [32], release of antimicrobial substances [33], decreased plaque formation rate [34,35,36,37], and changes in salivary secretion rate [38].

However, the use of probiotics in dentistry is still limited. One of the key problems is the lack of information on probiotics among both consumers and health care professionals (HCPs) [2,39,40]. Despite a growing number of studies showing the safety and efficacy of probiotics [21,27,41], the health professionals may be suspicious in recommending probiotics to patients when they receive conflicting reports [42]. Moreover, they may have difficulties in processing large volumes of information disseminated by commercial companies about the benefits of probiotics [42,43]. In addition, the efficacy of probiotics depends on the bacterial strain, dosage, and duration of administration [44]. The heterogeneity of these parameters in studies and other design features can lead to inconsistent results, thus not allowing clinicians to give evidence-based recommendations [45,46,47]. Previous studies have indicated that despite a positive attitude towards probiotics, most HCPs had fair knowledge of the topic [2,40,43]. The lack of knowledge of probiotics, along with the growing evidence supporting their efficacy, highlights the need for closer attention to the education of clinicians. Moreover, medical students, as future healthcare workers, have to know more about probiotics in order to give proper recommendations to their future patients [43]. University teachers play a major role in ensuring an appropriate level of knowledge among both students and clinicians, which ultimately will lead to better attitude and practices. To the best of our knowledge, no studies have evaluated the awareness of dental academics and university students regarding probiotic use. Such evaluation could reveal specific knowledge gaps and areas that need to be focused on through an educational process and provide details about possible barriers to the use of probiotics.

The aim of our study was to evaluate the knowledge of and attitude towards probiotics among dental students and academics.

## 2. Materials and Methods

### 2.1. Ethical Approval

The study was approved by Sechenov University’s Ethics Committee (Protocol no. 23-22, 17 November 2022).

### 2.2. Study Design, Target Population, and Sample Size

This cross-sectional, descriptive study was conducted between December 2022 and February 2023 and included dental students and teachers of the Therapeutic Dentistry Department (Sechenov University, Moscow, Russia).

The study’s total population consisted of 604 third-, fourth-, and fifth-year Russian-speaking and English-speaking dental students and 54 teachers, who were also practicing dentists.

A minimum sample size of 235 students and 48 teachers was calculated assuming a 95% CI and a margin of error of 5% using the following formula:

$$\mathrm{Sample}\mathrm{size}\;=\frac{\frac{z^{2}\times p\left(1-p\right)}{e^{2}}}{1+\left(\frac{z^{2}\times p\left(1-p\right)}{e^{2}N}\right)},$$

where

*z* (z-score, i.e., the number of standard deviations a given proportion deviates from the mean) = 1.96 (95% CI);
*p* (standard deviation) = 0.5;
e (margin of error) = 0.05;
*N* (population size) = 604 (students); 54 (teachers).

### 2.3. Questionnaire Development

There was no prior validated questionnaire to evaluate dental students’ and academics’ knowledge and attitude regarding probiotics. Thus, a questionnaire was developed by the authors in English and translated into Russian. The English version of the questionnaire was created based on the questionnaires used in previously published studies [2,48,49,50,51].

The expert panel consisting of a gastroenterologist, a dental professional, and a medical statistician approved the content validity of the questionnaire on both languages. In our pilot study, the face validity of the Russian version of the questionnaire was tested on a group of dental students (*n* = 30), whose feedback was used to eliminate words, phrases, or questions that were difficult to understand. The pilot study respondents were also asked to record the time they had spent on completing the questionnaire. Based on the feedback, the questionnaire was corrected by the authors to eliminate any ambiguities. The pilot sample data were excluded from the final analysis. Cronbach’s alpha for this questionnaire was not calculated, as each item represented a unique context rather than an underlying latent construct.

The final version of the questionnaire consisted of 15 questions divided into 3 sections: respondents’ sociodemographic data (5 questions) including age, sex, and residence; questions about participants’ subjective knowledge on probiotics (5 questions); and questions related to participants’ attitude towards probiotics (5 questions).

### 2.4. Data Collection and Analysis

The link to the online survey was sent via email and social media platforms. The survey was voluntary and anonymous and included no personal data. The respondents were informed that completion of the questionnaire indicated agreement to join the study and that their responses would be used for academic and research purposes. The data were collected through Google Form.

The knowledge section comprised one single-choice question, three multiple-choice questions, and one true or false question. Each correct answer was assigned a score of one, and each incorrect answer was assigned a score of zero. The maximum score for the knowledge questions was 22.

The five-point Likert scale was used to assess the four items of the attitude questions. Three items were positive attitude statements (5 = strongly agree, 4 = agree, 3 = neutral, 2 = disagree, 1 = strongly disagree), and one was a negative attitude statement (5 = strongly disagree, 4 = disagree, 3 = neutral, 2 = agree, 1 = strongly agree).

One more question was asked about the willingness of the respondents to recommend probiotics to patients in case their effectiveness was substantiated by peer-reviewed literature (3 = yes, 2 = not sure, 1 = no).

Thus, the maximum score for the attitude questions was 23.

The level of knowledge was categorized as good, fair, or poor. It was categorized as good if a respondent’s total score was >75% (17–22 points), fair if it was in the range of 50–75% (12–16 points), and poor if it was ≤50% (0–11 points) of the maximum score. The attitude scores were categorized as positive (total score ≥ 50% (12–23 points)) and negative (total score < 50% (5–11 points)).

Data entry was completed in the MS Excel database. The data were exported into the CSV file format, which was then used for data analysis in R version 3.6.0 (26 April 2019) with the following packages: “doBy”, “rstatix”, “stats”, and in RStudio version 1.2.1335 2009–2019. The data are presented as means, medians, standard deviations, 25th and 75th percentiles (quantitative variables), and counts and percentages (qualitative variables). The Mann–Whitney U test was performed to compare quantitative variables in the independent groups; the Fisher’s exact test was used to compare the proportions. The Spearman’s rank correlation coefficient was calculated to reveal the correlation between knowledge and attitude scores.

## 3. Results

**Figure 1:**
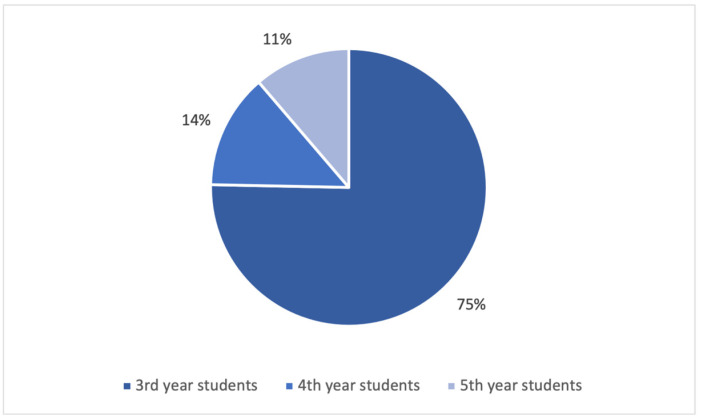
Students’ distribution by year of education.

Out of the 658 questionnaires distributed (604 to students and 54 to teachers), a total of 239 questionnaires were completed by the undergraduates, yielding a response rate of 39.6%, and 54 by the teaching staff (response rate = 100%). The response rate for 3rd-year students was higher than that for 4th- and 5th-year students (84.5%, 13.4%, and 17.8%, respectively, *p* < 0.001). Figure 1 shows the students’ distribution by year of education.

Table 1 displays the group, age, and sex distributions of the participants. A majority of the participants were females, accounting for 68.5% (*n* = 164) and 81.5% (*n* = 44) of the students and teachers, respectively. The mean age of the students was 21.5 years, and the mean age of the teachers was 39.8.

**Figure 2:**
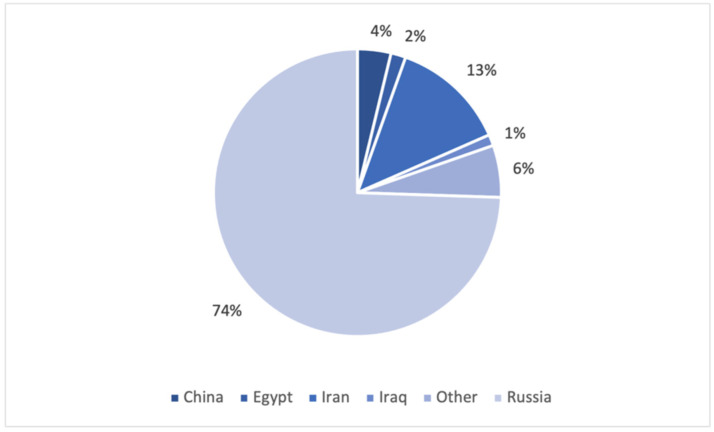
Students’ distribution by country of origin.

Most students were from Russia. Others were from countries including Iran, China, Iraq, Egypt, and others. The distribution of the respondents by country of origin is shown in Figure 2.

The majority of the students (53.6%) and teachers (55.5%) had a fair knowledge of probiotics. One in five University teachers (20.4%) demonstrated a good current understanding of the topic, whereas only 13.8% of undergraduates achieved a good knowledge score. A little over a third of the students (32.6%) had a poor level of knowledge. There were no significant differences between the groups regarding the knowledge of probiotics (Table 2).

Table 3 shows the distribution of the responses to the knowledge questions about probiotics across the study groups. The majority of the respondents in both groups were familiar with the term “probiotics” and defined it correctly. Concerning the health benefits linked to probiotics, approximately a fifth of undergraduates and a quarter of teachers chose all systemic conditions in which the use of probiotics may be beneficial. Only one clinician (1.9%) and eight students (3.3%) chose all the correct options regarding the bacterial species containing probiotic strains. The optimal time for probiotics intake was correctly chosen by 58.2% of the students and 55.5% of the academics.

**Figure 3:**
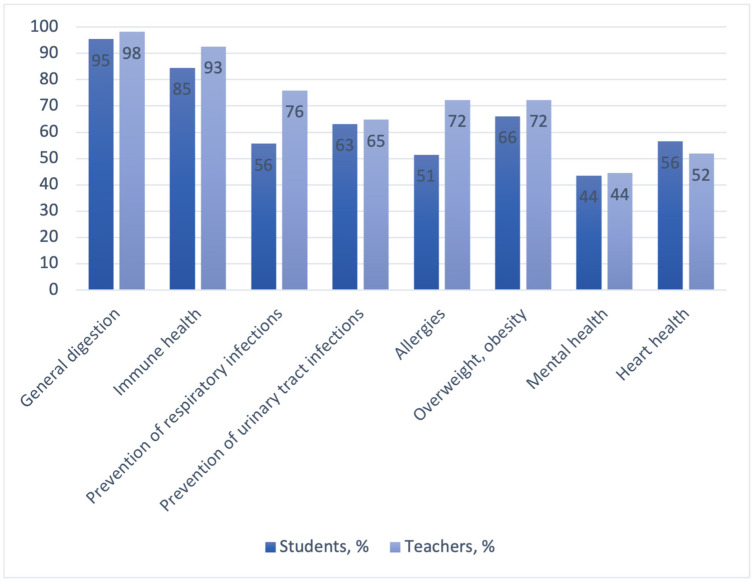
Respondents’ knowledge of probiotics’ benefits for health conditions.

A vast majority of the respondents in both groups knew that probiotics exert a favorable effect on gut health. Most respondents noted their role in immune health, in patients with obesity, and in the prevention of respiratory and urinary tract infections. The respondents in both groups were least aware of the probiotics benefits for mental and heart health (Figure 3).

**Figure 4:**
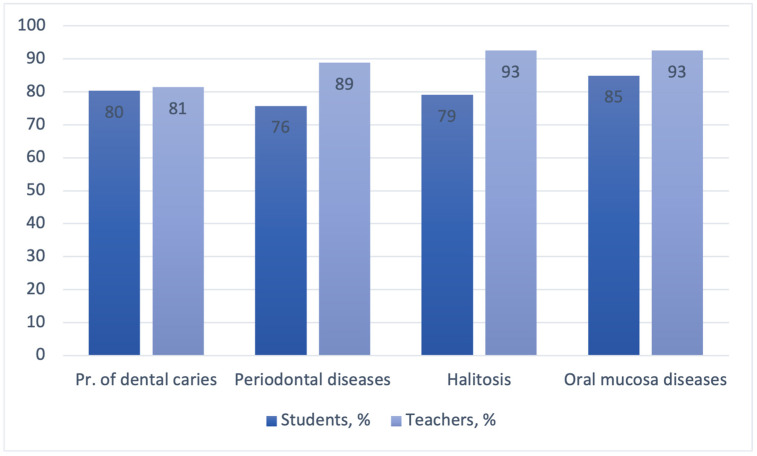
Respondents’ knowledge of probiotics’ benefits for oral health conditions.

Regarding oral health, more than half of the survey participants were aware of the role of probiotics in the prevention of dental caries and the treatment of halitosis and periodontal and oral mucosa diseases (Figure 4).

**Figure 5:**
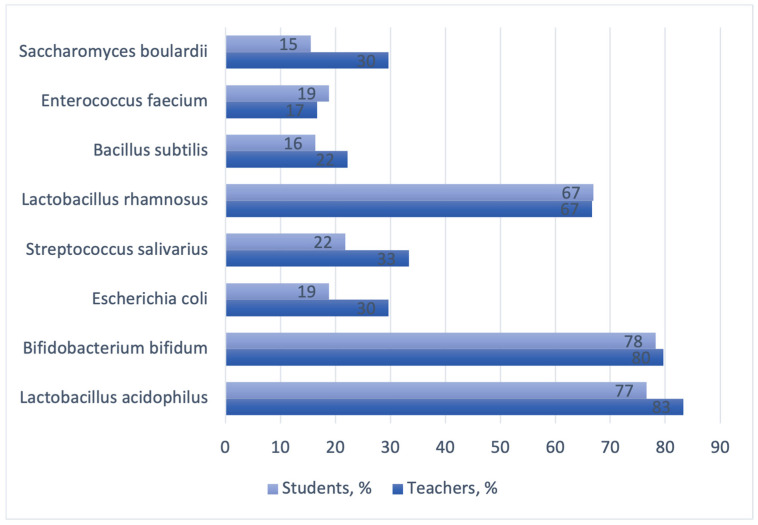
Respondents’ knowledge of bacterial species that include probiotic strains.

Out of the eight options, the most recognized bacterial species were *Lactobacillus acidophilus*, *Bifidobacterium bifidum*, and *Lactobacillus rhamnosus.* The bacterial species least recognized by the teachers were *Bacillus subtillis* (22%) and *Enterococcus faecium* (17%), while the students knew the least about the probiotic strains of *Saccharomyces boulardii* (19%), *Bacillus subtillis* (16%), and *Escherichia coli* (15%) (Figure 5).

A vast majority of dental students (97.9%) and all teachers had a positive attitude towards probiotics. The teachers reported a higher mean of the attitude scores compared to the undergraduates (*p* < 0.001) (Table 4).

More than a half of the students and academics agreed that probiotics are an evidence-based intervention for health and may be used in clinical medicine. Only five students (2.1%), and no teachers, strongly agreed that probiotics might be potentially dangerous. The majority of the undergraduate respondents and academics noted that there is a need for HCPs’ education on probiotics. Finally, the vast majority of teachers gave a positive answer about their willingness to recommend probiotics, if substantiated by peer-reviewed literature. Most students also answered this question positively (Table 5 and Table 6).

**Figure 6:**
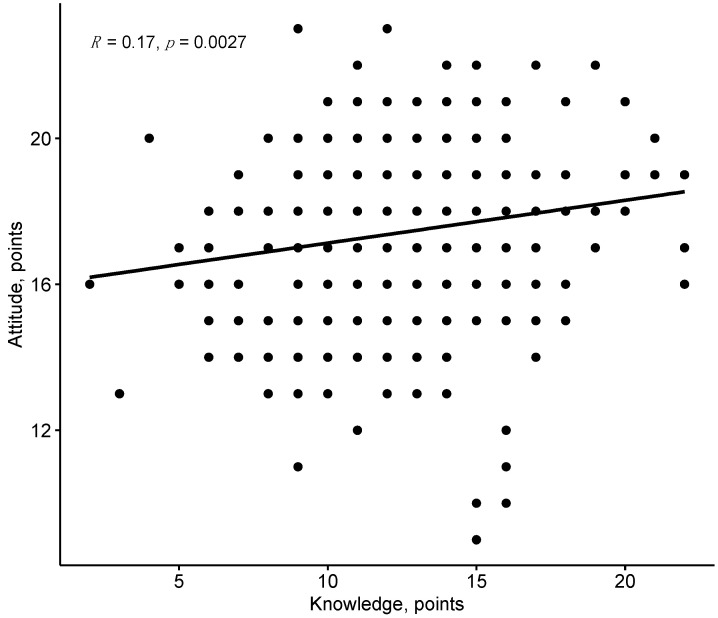
Correlation between knowledge and attitude.

Although weak, a positive correlation was found between knowledge and attitude (Spearman r = 0.17, *p* = 0.0027) (Figure 6).

## 4. Discussion

The balance between beneficial and pathogenic microorganisms is crucial for oral health [52,53,54]. Disturbance in this balance, called “dysbiosis”, may result in the development of oral and systemic diseases [55,56]. This makes the oral cavity a target for probiotics application [54]. Thus, dental practitioners should be aware of the application of probiotics in dentistry and their role in the prophylaxis and treatment of oral diseases. In this study, we aimed to assess the knowledge and attitude towards probiotics among dental students and teachers. Most of the respondents had a fair knowledge of probiotics, with no significant differences between the groups; nevertheless, a vast majority of the participants showed a positive attitude towards probiotics.

The study’s population included third-, fourth-, and fifth-year Russian-speaking and English-speaking dental students and teachers (Therapeutic Dentistry Department, Sechenov University). All teachers completed the survey (response rate = 100%). Interestingly, the response rate was significantly higher among the third-year students (84.5%) than among the fourth- (13.4%) and fifth-year (17.8%) students. This may be explained by the fact that younger students are better disciplined and have more spare time. Moreover, the third year is when our students start studying clinical subjects, which is why third-year students are generally more motivated to participate in activities related to their future practice.

We assessed the knowledge of probiotics in general and categorized it as “good”, “fair”, or “poor”, as well as the knowledge regarding particular questions, i.e., the definition of probiotics, their benefits for systemic and oral health, probiotic strains, and the favorable time of intake. This study showed that most of our respondents (53.6% of the students and 55.5% of the teachers) had a fair knowledge of probiotics. Only 13.8% of the undergraduates and 20.4% of the academics demonstrated a good knowledge of the topic. These results are in agreement with those of Payahoo et al. who found that 50.7% of medical students had a fair knowledge of probiotics [40]. In a study by Rahmah et al., most health science students had a fair (80%) or good (9.2%) knowledge of probiotics [43]. Arshad et al. surveyed HCPs on probiotics use and classified their knowledge as “good” or “poor” [2]. They found that only a small percentage of their respondents (15.1%) had a good knowledge of the topic.

In the present study, we found no significant differences between dental students and teachers regarding their knowledge of probiotics. In contrast, Soni et al. found that practitioners had a significantly higher level of awareness and knowledge of probiotic use compared to students [57]. Barqawi et al. concluded that although HCPs had a slightly better knowledge of the microbiota, it was not significantly better than that of non-HCPs, and almost half of the HCPs had average knowledge scores (49.4%) [58].

In our study, 77.4% of the students and 81.5% of the teachers defined the term “probiotics” correctly. This is in line with the results of other studies, in which the correct definition was chosen by over 80% of the respondents (students or HCPs) [40,42,43,50,57].

Only 21.3% of the dental students and 26% of the teachers in our study chose all systemic diseases in which the use of probiotics may be beneficial. In the study by Payahoo et al., about 60% of medical students identified the health benefits of probiotics correctly [40]. This may be due to a better knowledge of probiotics on the part of medical students in general compared to dental students. However, in the study at hand, more respondents (59.4% of undergraduates and 70.4% of academics) were aware of the role of probiotics in the prevention and treatment of oral diseases.

According to the studies by Fijan et al. and Rahmah et al., *Bifidobacterium* and *Lactobacillus* were the most recognizable genes of bacteria containing probiotic strains [42,43]. Our data supports the evidence from the aforementioned studies. Out of the eight options, the most recognized bacterial species in both groups were *Lactobacillus acidophilus*, *Bifidobacterium bifidum*, and *Lactobacillus rhamnosus*. These bacterial genera are most commonly used in the prophylaxis and treatment of oral diseases [53].

We found that the bacterial species least recognized by the teachers included *Bacillus subtillis* (22%) and *Enterococcus faecium* (17%), while the students knew the least about the probiotic strains *Saccharomyces boulardii* (19%), *Bacillus subtillis* (16%), and *Escherichia coli* (15%). Similarly, *Enterococcus faecium*, *Saccharomyces boulardii,* and *Escherichia coli* were correctly listed as containing probiotic strains in the study by Fijan et al. by less than a third of the respondents [42].

Over 50% of the students and academics claimed that probiotics should be taken before meals. While it is widely accepted, the literature addressing this question is scarce, and more studies are necessary to make correct claims. We considered the answer “before meal” as correct in accordance with a study by Tompkins et al. [59].

In this study, a vast majority of the respondents in both groups had a positive attitude towards probiotics. Interestingly, the mean attitude score was significantly higher among the academics than among the students. Despite a positive attitude in general, almost 30% of the students “strongly agreed” or “agreed” that probiotics may be dangerous. At the same time, only 7.4% of the teachers “agreed” with this statement. Around 80% of the respondents in both groups noted the importance of additional education on probiotics among HCPs. In many previous surveys, the majority of HCPs demonstrated a positive attitude regarding the use of probiotics [2,50,57,60]. However, in the study by Rahmah et al., only a half of health science students had a positive attitude towards their use [43].

Doctors are expected to prescribe drugs using an evidence-based approach [61]. The last question in the “Attitude” section was about the readiness to recommend probiotics if their effectiveness was substantiated by peer-reviewed literature. Surprisingly, 5% of the students were not ready to administer probiotics even if their effect was evidence-based, and 16% of the students had doubts about this question. In the study by Rahmah et al., the proportion of the students unwilling to recommend probiotics was even higher: more than a half had a neutral position, and 10% of them did not intend to use probiotics in their practice [43]. On the other hand, a vast majority of academics in our study answered the question positively. Similarly, Basrowi et al. reported that 97% of HCPs (pediatricians) considered prescribing probiotics to their patients [62]. In a study by Oliver et al., 77% of health care providers were “quite a bit” and “very much” willing to recommend probiotics if their effectiveness was substantiated by peer-reviewed literature [50]. According to Fijan et al., the majority of medical doctors (82.4%) and dentists (83.3%) had advised their patients to use probiotics [42].

It is important to note that there are different regulations on prescribing probiotics in different countries [42]. However, the negative attitude of the students toward probiotics administration in the present study cannot be attributed to any restrictions, as the majority of the respondents were from countries where the prescription of probiotics is not prohibited.

In our study, a weak yet positive correlation was found between knowledge and attitude scores. The results obtained corroborate the findings from the study by Rahmah et al. [43]; however, they differ from the findings presented by Soni et al. These authors showed that there was a positive and significant correlation between the knowledge and the attitude of HCPs [57].

## 5. Limitations

The present study included academics and students from one university, which is why its results cannot be generalized. However, since the survey was conducted in the largest medical university of Russia, with an extensive international student population, we expect the results to be representative. Another factor that may have affected the results is that we used convenience sampling and a population comprising mostly third-year university students. In addition, another limitation is that practice was not assessed. A response bias may be present due to the use of self-reporting data, which may affect the accuracy of the findings. Moreover, the use of the Likert scale instead of open questions could have influenced the results.

## 6. Conclusions

The results of our survey showed a fair knowledge of probiotics among dental students and teachers. The main knowledge gaps revealed include the effects of probiotics on systemic and oral health, bacterial species that comprise probiotic strains, and the regimen of probiotics intake. At the same time, the participants demonstrated a positive attitude towards the use of probiotics. To translate their positive attitude into practice and raise awareness regarding the use of probiotics, there is a need for more evidence-based educational trainings for university teachers, who play an important role in educating medical students and clinicians. A course on probiotics focused on the aforementioned knowledge gaps should be included in the curriculum of dental students.

## Figures and Tables

**Table 1:** Subject demographics.

	Students	Teachers
**Sex** n (%) ^1^		
Female	164 (68.6)	44 (81.5)
Male	75 (31.4)	10 (18.5)
Total	239 (100)	54 (100)
**Age**		
Mean (SD)	21.5 (2.4)	39.8 (11.0)
Median [Q1, Q3]	21 [20, 22]	37 [30, 50.75]
Min, Max	18, 38	24, 60

SD—standard deviation; ^1^ difference between the groups was insignificant (0.0683) according to Fisher’s exact test.

**Table 2:** Knowledge of probiotics among dental students and teachers.

	Knowledge Abs (%)			*p*-Value ^1^	Mean ± SD	*p*-Value ^2^
	Poor	Fair	Good			
Students	78 (32.6)	128 (53.6)	33 (13.8)	0.3135	13.0 ± 3.6	0.1594
Teachers	19 (24.1)	30 (55.5)	11 (20.4)		13.7 ± 3.3	

Abs—absolute number; SD—standard deviation; ^1^ according to Fisher’s exact test; ^2^ according to Mann-Whitney U test.

**Table 3:** Distribution of the responses to the knowledge questions about probiotics among dental students and teachers.

Question	Students, Abs (%)		Teachers, Abs (%)		*p*-Value ^1^
	CA	IA	CA	IA	
Definition of the term	185 (77.4)	54 (22.6)	44 (81.5)	10 (18.5)	0.5876
Probiotic benefits for systemic diseases *	51 (21.3)	188 (78.7)	14 (26.0)	40 (74.0)	0.4712
Probiotic benefits for oral diseases *	142 (59.4)	97 (40.6)	38 (70.4)	16 (29.6)	0.1639
Bacterial species that have probiotic strains *	8 (3.3)	231 (96.7)	1 (1.9)	53 (98.1)	1.0
Optimal time for probiotics intake	139 (58.2)	100 (41.8)	30 (55.5)	24 (44.4)	0.7616

Abs—absolute number; CA—correct answer; IA—incorrect answer; * respondents chose all correct answers; ^1^ according to Fisher’s exact test.

**Table 4:** Respondents’ attitude toward probiotics.

	Attitude Abs (%)		*p*-Value ^1^	Mean ± SD	*p*-Value ^2^
	Positive	Negative			
Students	234 (97.9)	5 (2.1)	0.5883	17.1 ± 2.3	<0.001
Teachers	54 (100.0)	0 (0)		19.1 ± 2.0	

Abs—absolute number; SD—standard deviation; ^1^ according to Fisher’s exact test; ^2^ according to Mann–Whitney U test.

**Table 5:** Distribution of the responses to the attitude questions about probiotics among dental students.

Item	Respondents’ Answers Abs (%)				
	SA	A	N	D	SD
Probiotics may be used in clinical medicine	43 (18.0)	123 (51.5)	52 (21.7)	14 (5.9)	7 (2.9)
Probiotics are an evidence-based intervention for health	29 (12.1)	123 (51.5)	62 (25.9)	22 (9.2)	3 (1.3)
Probiotics can be dangerous for health	7 (2.9)	62 (26.0)	82 (34.3)	76 (31.8)	12 (5.0)
There is a need for healthcare professional education on probiotics	55 (23.0)	128 (53.6)	38 (16.0)	13 (5.4)	5 (2.0)
	Yes		Not sure		No
If substantiated by peer-reviewed literature, would you be willing to recommend probiotics to your patients?	188 (78.7)		38 (15.9)		13 (5.4)

Abs—absolute number; SA—strongly agree; A—agree; N—neutral; D—disagree; SA—strongly disagree.

**Table 6:** Distribution of the responses to the attitude questions about probiotics among dental teachers.

Item	Respondents’ Answers Abs (%)				
	SA	A	N	D	SD
Probiotics may be used in clinical medicine ^1^	23 (42.6)	22 (40.7)	6 (11.1)	2 (3.7)	1 (1.9)
Probiotics are an evidence-based intervention for health ^2^	11 (20.4)	30 (55.6)	10 (18.5)	3 (5.5)	-
Probiotics can be dangerous for health ^3^	-	4 (7.4)	7 (13.0)	33 (61.1)	10 (18.5)
There is a need for healthcare professional education on probiotics ^4^	16 (29.6)	29 (53.7)	9 (16.7)	-	-
	Yes		Not sure		No
If substantiated by peer-reviewed literature, would you be willing to recommend probiotics to your patients? ^5^	52 (96.3)		2 (3.7)		-

Abs—absolute number; SA—strongly agree; A—agree; N—neutral; D—disagree; SA—strongly disagree; ^1^ *p* = 0.005214; ^2^ *p* = 0.3902; ^3^ *p* < 0.001; ^4^ *p* = 0.3397; ^5^ *p* = 0.005857, according to Fisher’s exact test.

## Data Availability

The datasets used and/or analyzed during the current study are available from the corresponding author upon reasonable request.
